# Modular endolysin of *Burkholderia* AP3 phage has the largest lysozyme-like catalytic subunit discovered to date and no catalytic aspartate residue

**DOI:** 10.1038/s41598-017-14797-9

**Published:** 2017-11-06

**Authors:** Barbara Maciejewska, Karol Źrubek, Akbar Espaillat, Magdalena Wiśniewska, Krzysztof P. Rembacz, Felipe Cava, Grzegorz Dubin, Zuzanna Drulis-Kawa

**Affiliations:** 1 Institute of Genetics and Microbiology, University of Wroclaw, Przybyszewskiego 63/77, 51-148 Wroclaw, Poland; 2 Department of Microbiology, Faculty of Biochemistry, Biophysics and Biotechnology, Jagiellonian University, Gronostajowa 7, 30-387 Kraków, Poland; 3 Laboratory for Molecular Infection Medicine Sweden. Molecular Biology Department, Umeå University, SE-901 87 Umeå, Sweden; 4 Protein Crystallography Research Group, Malopolska Centre of Biotechnology, Gronostajowa 7A, 30-387 Krakow, Poland

## Abstract

Endolysins are peptidoglycan-degrading enzymes utilized by bacteriophages to release the progeny from bacterial cells. The lytic properties of phage endolysins make them potential antibacterial agents for medical and industrial applications. Here, we present a comprehensive characterization of phage AP3 modular endolysin (AP3gp15) containing cell wall binding domain and an enzymatic domain (DUF3380 by BLASTP), both widespread and conservative. Our structural analysis demonstrates the low similarity of an enzymatic domain to known lysozymes and an unusual catalytic centre characterized by only a single glutamic acid residue and no aspartic acid. Thus, our findings suggest distinguishing a novel class of muralytic enzymes having the activity and catalytic centre organization of DUF3380. The lack of amino acid sequence homology between AP3gp15 and other known muralytic enzymes may reflect the evolutionary convergence of analogous glycosidases. Moreover, the broad antibacterial spectrum, lack of cytotoxic effect on human cells and the stability characteristics of AP3 endolysin advocate for its future application development.

## Introduction

Muralytic enzymes are peptidoglycan (PG) degrading proteins widely represented within bacteriophages (bacterial viruses), bacteria, archaea, and eukaryotes. Among bacteriophages, muralytic enzymes may occur as virion-associated lysins serving for PG degradation at the first step of host infection, or as endolysins required at the end of a viral lytic cycle to allow progeny release from the infected cell^1–3^. Intensive research has been conducted in the recent years on antibacterial properties of phage endolysins. *In vitro* studies and in *in vivo* investigation in animal models, recently reviewed by Nelson and co-workers^4^, demonstrated that these enzymes are highly effective in the eradication of Gram-positive pathogens such as *Streptococcus pneumoniae*, *Staphylococcus aureus*, *Bacillus anthracis* and *Enterococcus faecalis*. In contrast, endolysins have a limited activity against Gram-negatives because of their impermeable outer membrane (OM) which protects the bacterial cell wall from exogenous PG-degrading enzymes. Only a few endolysins were proved to be exogenously effective against Gram-negative bacteria^5–7^. Nevertheless, recent studies have reported the effectiveness of modified endolysins or enzymes combined with membrane-permeabilizing agents to overcome OM barrier and destroy bacterial cells^8–11^.

The vast majority of known endolysins derived from Gram-negatives infecting phages are 15–20 kDa proteins containing a single enzymatic domain of simple globular structure. Rare examples of modular endolysins are usually composed of a single N-terminal cell wall-binding domain (CBD) responsible for high-affinity tying to the cell wall receptor and a C-terminal enzymatically active domain (EAD) responsible for breaking specific bonds within the PG structure^4^. Modular architecture has been demonstrated for several endolysins produced by Gram-negatives infecting phages: KZ144, EL188, OBPgp279 and 201φ2-1gp229 encoded by *Pseudomonas* phages^12,13^ and PVP-SE1gp146, SPN1S_0028, Lys68 and Gp110 encoded by *Salmonella* phages^10,13–15^.

The cleavage specificity of muralytic enzymes (including endolysins) falls into three major categories: amidases, endopeptidases, and glycosidases, depending on the type of the chemical bond that is cleaved within PG. Amidases hydrolyse the amide bond between the sugar and the peptide moieties and endopeptidases cleave the PG within the peptides^4^. The major group, however, consists of glycosidases, which cleave one of the two glycosidic bonds in the glycan chain, and are subdivided into glucosaminidases, lysozymes (muramidases) and lytic transglycosylases^3^. Glucosaminidases hydrolase *β*-1,4–glycosidic bond between N-acetylglucosamine (Glc*N*Ac) and N-acetylmuramic acid (Mur*N*Ac) on the reducing side of Glc*N*Ac. Lysozymes and lytic transglycosylases cleave the same bond, but on the reducing side of Mur*N*Ac, wherein lysozymes simply hydrolase the *β*-1,4–glycosidic bond and lytic transglycosylase release a product containing 1,6-anhydro ring at the Mur*N*Ac residue^16^. Lysozymes are the most common muralytic enzymes. Based on the fold similarity, four general families of lysozymes with demonstrated PG-hydrolytic activity have been described so far: (i) hen egg-white lysozyme (HEWL), (ii) goose egg-white lysozyme (GEWL), (iii) bacteriophage T4 lysozyme (T4L) and (iv) *Chalaropsis* lysozyme^17^. A characteristic feature of lysozymes is their great diversity in terms of the amino-acid sequence. HEWL, GEWL and T4L lysozymes share closest similarities in three-dimensional structure and catalytic centre organization. All share the same characteristic set of hydrogen bonds between the backbone of the enzyme and the 2-acetamido group of the saccharide in subsite C. HEWL catalytic centre is located in a crevice between two sub-domains, which are connected by a long α-helix. In a vast majority of lysozymes, two residues: a general-acid catalyst residue - glutamic acid (Glu, E) and a general-base catalyst residue - aspartic acid (Asp, D) or cysteine (Cys, C) are involved in catalysis. Besides the Glu and Asp/Cys, the third accessory and catalytically important residue – threonine (Thr) or serine (Ser) may take part in the catalytic reaction^18^. Thr/Ser as a catalytic water positioning residue (in the sequence of Glu-8aa-Asp/Cys-5aa-Thr catalytic triad) was previously demonstrated for lysozymes of coliphages T4 and P21 (Glu11-Asp20-Thr26 and Glu35-Asp44-Thr50 respectively)^18,19^. GEWL lysozymes are an exception having only a single catalytic residue – Glu^20^. Despite the differences in the cleavage products, the single catalytic residue (Glu) is also characteristic for lytic transglycosylases including phage lambda endolysin^17^.

The initial classification of the activity type of new enzymes is often based on *in silico* homology. It is therefore hampered if a large sequence diversity is encountered in a single class, particularly with a limited number of well-defined canonical members^21^. Currently, large sets of uncharacterized protein families are still found in databases. Indeed, ca. 1,600 domains of unknown function (DUFs) exist in Pfam protein database. A wide distribution of certain DUFs among different organisms suggests their evolutionary importance. Based on HMMER reference proteome database the number of DUF3380 domain homologues is currently estimated at 233 (209 bacteria, 23 viruses, and 1 eukaryote). Therefore, it is crucial to determine the structure and catalytic activity of this family representative to guide future development in the field.

In this study, we present a comprehensive characterization of recombinant AP3gp15 endolysin encoded by the recently described phage AP3 (vB_BceM_AP3), specific to multidrug-resistant *Burkholderia cenocepacia* ^22^. This modular endolysin is composed of CBD domain and EAD domain classified by BLAST into DUF3380 family. In this study, we describe its crystal structure, cleavage specificity, antibacterial activity and spectrum, as well as stability and cytotoxicity to human cells. It is the first report describing the three-dimensional structure of DUF3380 domain combined with the correlation analysis of enzymatic function. Thus, our study provides a general structural and functional description of over two hundred DUF3380 homologues.

## Results

### *In silico* analysis of AP3 endolysin

**Figure 1:**
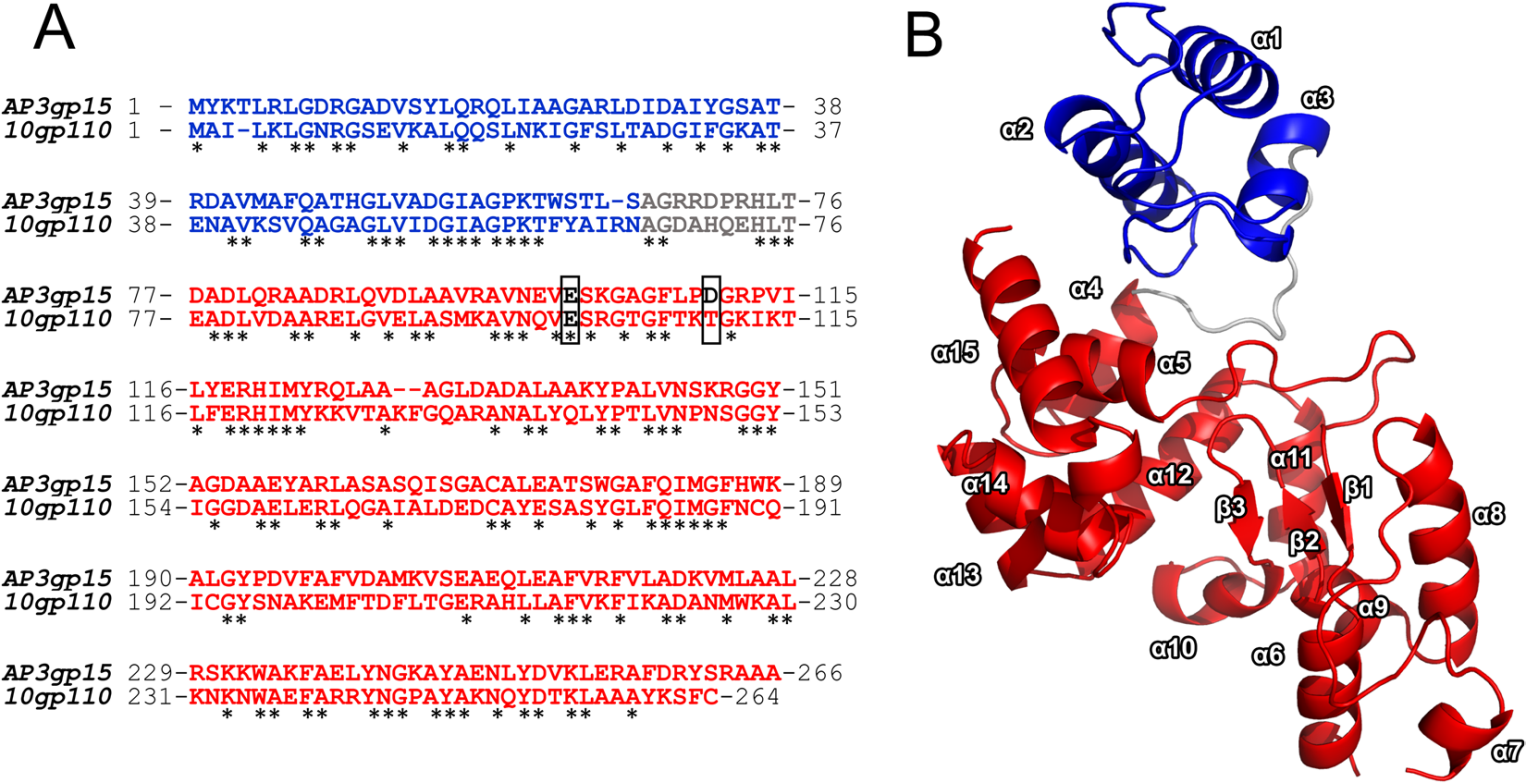
The modular structure of AP3gp15: blue, grey and red colours represent the CBD, hinge region and EAD (DUF3380), respectively. (**A**) The amino acid sequence comparison of AP3gp15 and 10gp110: the active residue Glu (E) at the position 101 is in black and framed; the Asp (D) at the position 110 in AP3gp15 (black) and its equivalent Thr (T) in 10gp110 (red) are framed. (**B**) Two asymmetrical domains are visible, CBD (N-terminal bundle of three helices representing the putative PG binding domain) encompassing residues Lys3 – Ser66; the catalytic domain EAD (residues Asp77 – Ala264); the hinge region (residues Ala67 to Thr76) connects both subunits.

Phage AP3, a representative of Gram-negatives infecting phages, utilizes a complex of four proteins which ensures efficient progeny release from an infected host cell. Its endolysin (AP3gp15), together with antiholin (AP3gp13), holin (AP3gp14) and bimolecular spanin (AP3gp16 and AP3gp17) form the lysis cassette^22^. AP3gp15 is a 266-amino acid protein with molecular mass of 28.9 kDa and theoretical pI of 8.82. The structure of endolysin, predicted *in silico* in BLASTP, is modular, consisting of CBD (10-65 aa; PG_binding_1 domain; pfam01471) at the N-terminus, and DUF3380 domain (pfam11860) at the C-terminus (90-262 aa) (Fig. 1A). There are at least 73 known significant homologues to modularly organized AP3gp15 and 37 homologues with reversely arranged domains. There are also 2428 and 233 homologues (E-value < 0.0001) of N-terminal CBD and C-terminal EAD, respectively, if compared separately. Domains similar to AP3gp15 CBD have been already well characterized in terms of the structure and function^23,24^, however, structural information concerning DUF3380 is non-existent. Recently, the gp110 endolysin derived from *Salmonella* phage 10, possessing DUF3380 has been analysed regarding its cleavage specificity and stability features^15^. However, the amino acid identity between phage 10 gp110 and AP3gp15 is less than 50% (46% for the whole protein sequence and 49% for CBD and 45% for EAD, if compared separately) (Fig. 1A). Moreover, both proteins differ significantly in terms of activity strength and stability features. Referring to above differences, the determination of the cleavage specificity and stability of AP3gp15 had to be examined (see below).

### The modular structure of AP3 endolysin

**Figure 2:**
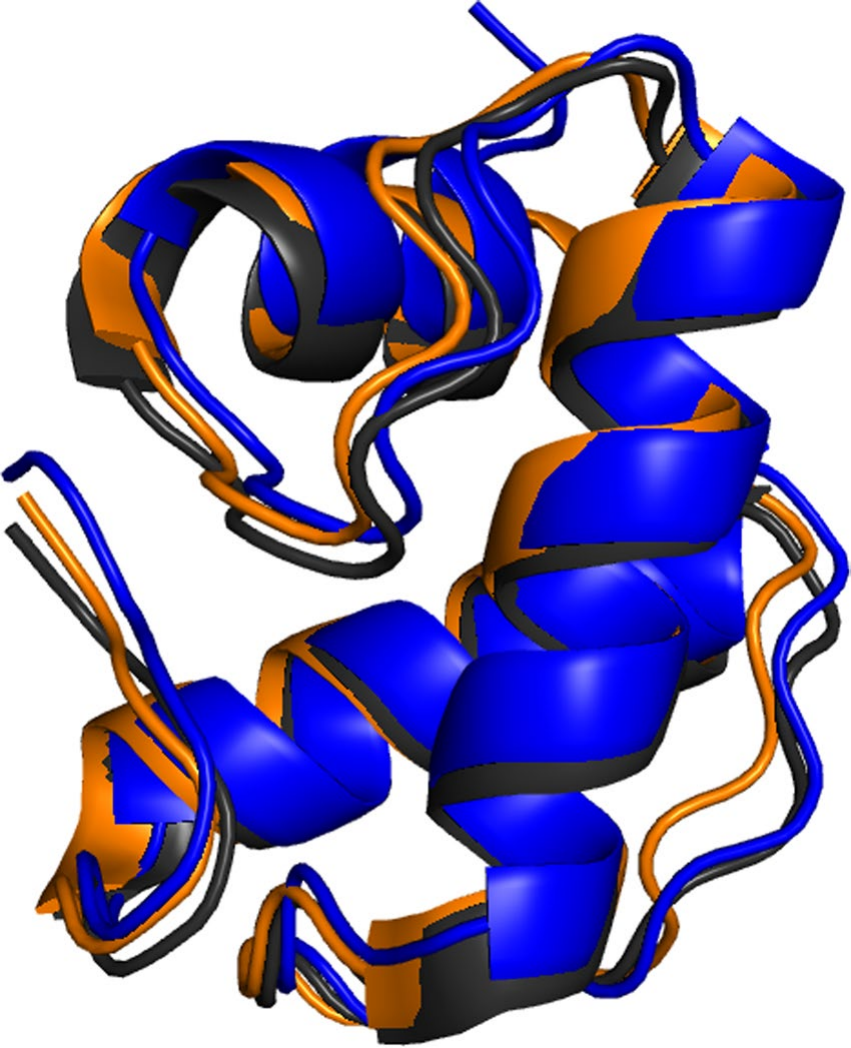
Structural alignment of CBD domains of AP3gp15 (blue), phage phiKZ endolysin (orange) and Zn-dependent amidase from *C*. *acetobutylicum* (grey) highlights a strong conservancy of three antiparallel helices bundle motif.

To provide the structural basis of the catalytic activity of DUF3380, the X-ray crystal structure of AP3gp15 has been evaluated (Fig. 1B; Table 1). The overall structure comprises of two clearly distinguished domains, a smaller N-terminal CBD domain (Lys3 – Ser66), and a larger EAD domain (Asp77 – Ala264) located towards the C-terminus (Fig. 1B). CBD domain is composed of a bundle of three antiparallel helices (α1-α3) and is joined with EAD *via* a hinge region spanning amino acids (Ala67 – Thr76). The EAD domain is organized into two sub-domains: first encompassing helices α4-α5 (Asp77 – Glu101) and helices α10 to α15 (Gly185 – Ala266), and the second containing β1 (Ile115 – Tyr117), β2-β3 (Thr176 – Met184) sheet intervened by helices α6 to α9 (Arg119 – Ala175). The structure of the EAD domain of AP3gp15 is characterized by a deep concave cleft at the subdomain interface. In order to identify the likely functional role of domains within AP3gp15 with particular emphasis on EAD, we compared them with all known structures within PDB using algorithms implemented in the Dali server^25^. The N-terminal domain demonstrated significant structural homology to PG binding domains (CBDs) found in other endolysins and multiple functionally distant proteins. The closest structural homologues, include respective domains of Zn-dependent amidase from *Clostridium acetobutylicum* (PDB ID 4XXT; RMSD = 1.1 for 65 C_α_ atoms (95%))^26^ and *Pseudomonas aeruginosa* phage phiKZ gp144 endolysin (PDB ID 3BKH; RMSD = 1.1 for 65 C_α_ atoms (94%))^24^ (Fig. 2). Despite a relatively low homology of amino acid sequence (31% and 39% identity, respectively), a high structural homology of the entire AP3gp15 N-terminal part to the domains of clearly identified function strongly suggests its role in PG interaction. The surface residue analysis of CBD, similarly to homologues, did not reveal any distinct negatively or positively charged patches. However, surface amino acids (Arg6, Arg10, Asp13, Asp31, and Asp54) were spatially homologous to Arg5, Arg9, Asp11, Asp31 and Asp 54 of Zn-dependent amidase, respectively. Also, the residues of AP3gp15: Arg6, Asp13, Asp31, Asp40, Asp54 have their equivalents in phage phiKZ endolysin residues: Lys58, Asp65, Asp83, Asp91, and Asp106. The most common fingerprint between those three proteins comprises of Arg6, Asp31, and Asp54 of AP3gp15 CBD. The conservative character of residues likely suggests their role in PG recognition and binding.

**Table 1:** Data collection and refinement statistics.

	**Native**	**SeMet** (**peak**)
**PDB ID**	5NM7	—
**Data collection**		
Space group	H3_2_	P12_1_1
Unit cel dimensions		
a,b,c (Å)	154.62, 154.62, 125.82	67.10, 94.02, 95.80
α,β,γ (°)	90, 90, 120	90, 96.14, 90
Resolution (Å)	26.72-1.72 (1.75-1.72)	47.62-2.42 (2.50-2.42)
Observed reflections	553,404 (28,496)	309,340 (29,038)
Unique reflections	61,070 (3,214)	45,239 (4,455)
R_merge_ (%)	12 (64.3)	7 (61.9)
Completeness (%)	100 (100)	99.9 (99.9)
I/σI	11.1 (3.3)	18.2 (2.6)
Multiplicity	9.1 (8.9)	6.8 (6.5)
**Refinement**		
Resolution (Å)	26.72-1.72	
R_all_ (%)	20.84	
R_free_ (%)	23.53	
R_msd_ from ideal values		
Bond length (Å)	0.0113	
Bond angles (°)	1.4028	
Average B factors (Å^2^)	29.8	
Wilson plot B factors (Å^2^)	33.8	
Ramachandran statistics		
Most favoured regions	98.43	
Allowed regions	1.57	
Content of asymmetric unit		
No. of protein molecules	2	
No. of protein residues/atoms	524/4207	
No. of solvent atoms	180	
No. of ligands	6

**Figure 3:**
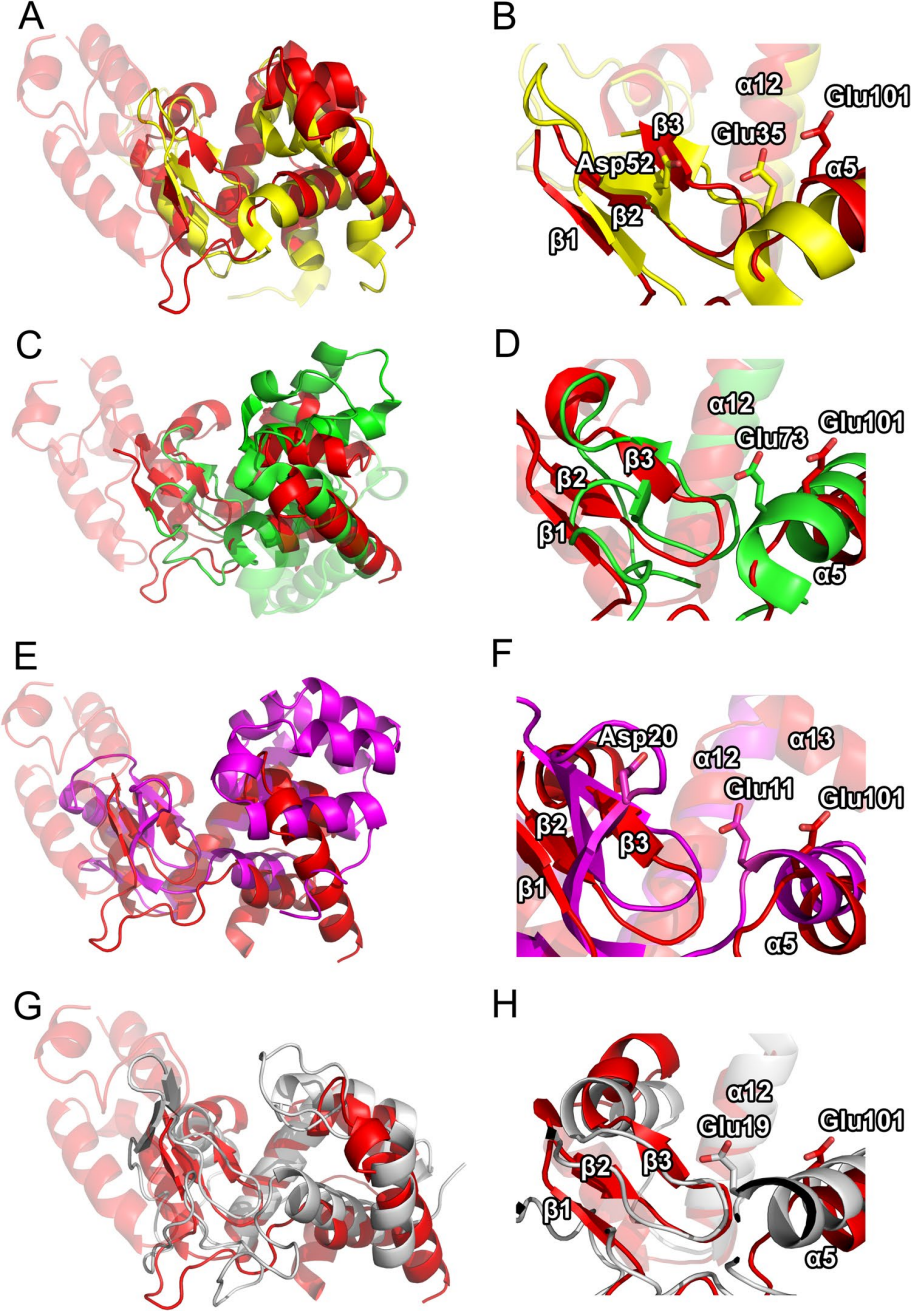
Structural alignment of AP3gp15 DUF3380 domain (red) and: (**A**) HEWL (yellow); (**C**) GEWL (green); (**E**) T4 lysozyme (purple) and (**G**) phage lambda lytic transglycosylase (silver). Structural alignment of the catalytic center of the AP3 endolysin DUF3380 domain (red) and: (**B**) HEWL (yellow); (**D**) GEWL (green); (**F**) T4 lysozyme (purple) and (**H**) phage lambda lytic transglycosylase (silver).

The large enzymatic C-terminal domain of AP3 endolysin shows structural homology to proteins of lysozyme family. HEWL, GEWL, bacteriophage T4 lysozyme and phage lambda lysozyme have been used for further comparison due to the detailed structure-function relationship data available for those proteins. The structural similarity of AP3gp15 to HEWL (PDB ID 4HPI)^27^ (rmsd~3.3 Å over 128 residues: 12% identity, Z-factor of 5.1), GEWL (PDB ID 153 L)^20^ (rmsd~3.7 Å over 185 residues: 8% identity, Z-factor of 5.1), T4L (PDB ID 1LYD)^28^ (rmsd~3.8 Å over 164 residues: 10% identity, Z-factor of 2.3), and lambda endolysin (PDB ID 1D9U)^29^ (rmsd~3.8 Å over 154 residues: 15% identity, Z-factor of 5.7) has been established by Dali server algorithm. Despite the low similarities, AP3gp15 reveals several common structural components with HEWL, GEWL, T4L, lambda endolysin (Fig. 3A,C,E,G). The comparison to HEWL, the closest structural homologue among lysozymes, allowed to define the core structure of AP3 endolysin, where the entire fold of HEWL is encompassed within α5, α10, α12, α14 and α15. This core is decorated with large protrusions (α6-α9) characteristic only for AP3 phage endolysin and not present in classical lysozymes (Fig. 3A).

### Structural homology suggests an unusual catalytic mechanism of the AP3gp15 enzymatic domain

**Figure 4:**
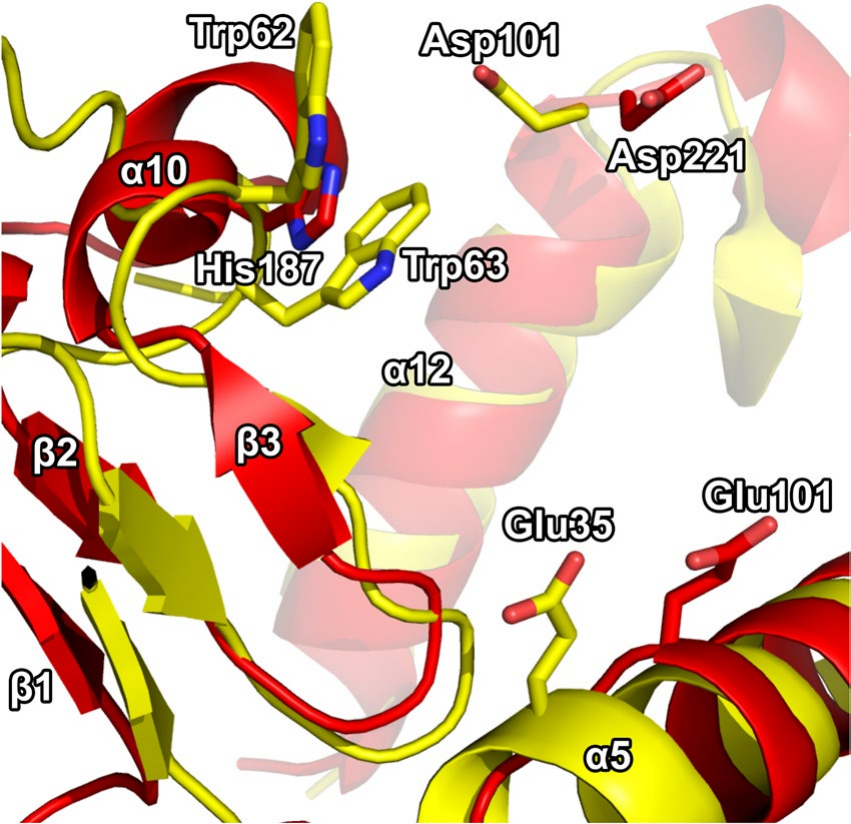
Structural alignment of AP3gp15 and HEWL loop region responsible for substrate recognition. Homologies between HEWL and AP3gp15 residues that are possibly involved in the substrate recognition and other interactions. Only the homologous catalytic Glu35 of HEWL is highlighted as it is represented by its counterpart residue of Glu101 in AP3gp15. Asp101 of HEWL plays a role in substrate positioning and its putative homologue is Asp221 of AP3gp15. The closest homologue of similarly functioning Trp62 and Trp63 of HEWL is represented by His187 that positions between these two amino acids in the structural alignment.

The structure alignment of AP3 endolysin to HEWL, GEWL, T4 lysozyme and lambda lytic transglycosylase suggests the probable catalytic center elements interacting with the substrate (Fig. 3B,D,G,H). Glu35 and Asp52 of HEWL, Glu73 of GEWL, Glu11 and Asp20 of T4L, and Glu19 of lambda endolysin have been identified as the key residues involved in the catalytic process^29,30^. AP3gp15 contains only Glu equivalent at the presumed catalytic centre (position 101). No Asp equivalent, as the second catalytic residue, could have been identified in the active center of AP3gp15.The substrate is most likely recognized by residues located in α5, α10, a12, α14 helices and a loop region (Ser102 – Val114) of AP3gp15. In particular, Asp221 and His187 as homologues of Asp101 and Trp62-63 (involved in recognition and binding of PG sugar residues by HEWL^27^) were identified as taking part in the binding of the oligosaccharide substrate (Fig. 4).

### AP3 endolysin has lysozyme specificity

**Figure 5:**
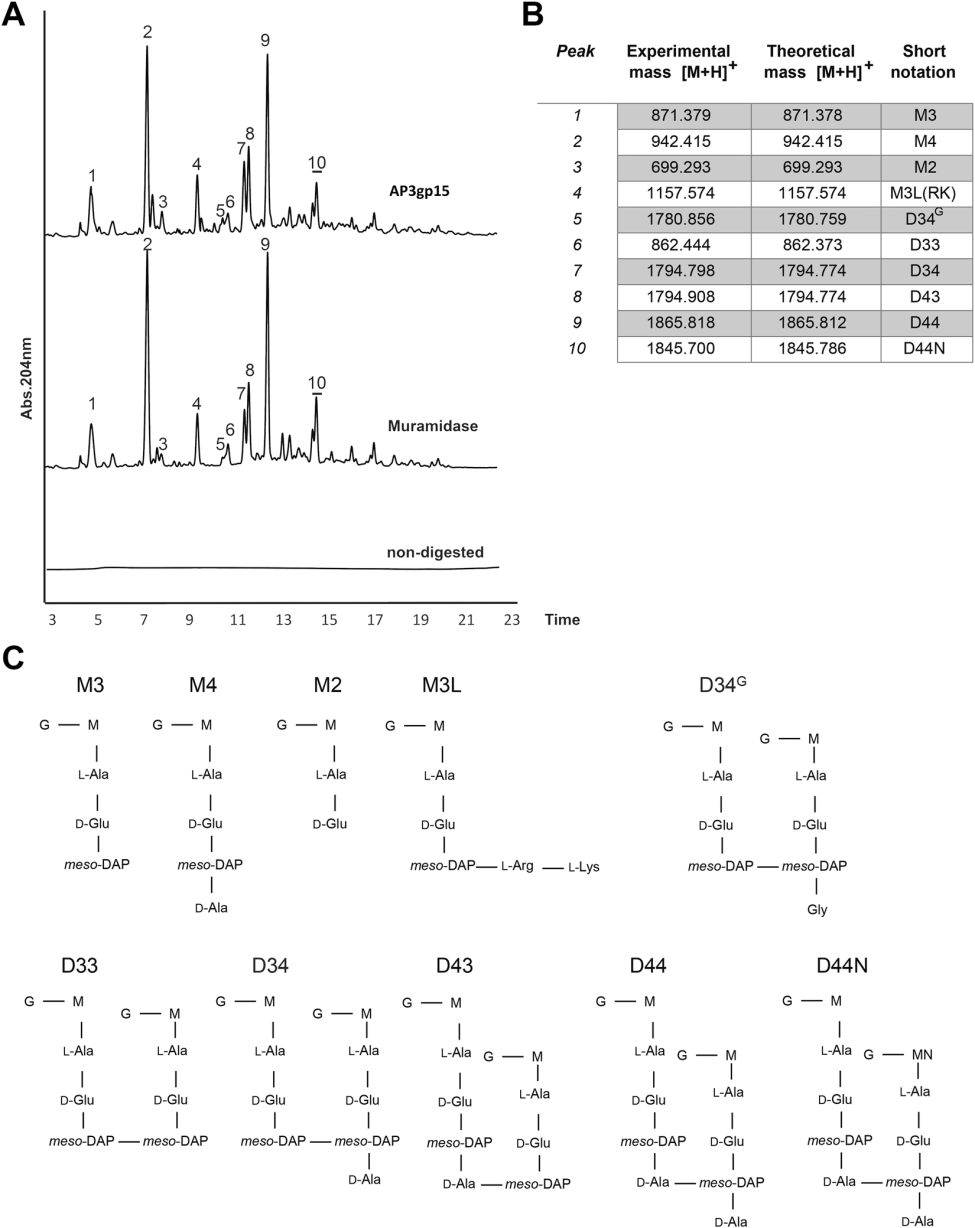
Cleavage specificity of AP3gp15. (**A**) UPLC chromatogram of *in vitro* digestion of *E*. *coli* sacculus either with muramidase, AP3gp15 or without enzyme (non-digested). (**B**) Mass spectrometry muropeptide analysis. **M2**, N-acetylglucosamine-(Glc*N*Ac)-N-acetylmuramic acid (Mur*N*Ac)-l-Ala-d-Glu; **M3**, Glc*N*Ac-Mur*N*Ac-l-Ala-d-Glu-meso-DAP; **M4**, GlcNac-Mur*N*Ac-l-Ala-d-Glu-meso-DAP-d-Ala; **M3L**, Glc*N*Ac-Mur*N*Ac-l-Ala-d-Glu-meso-DAP-Arg-Lys, oligopeptide from Braun’s lipoprotein which remains bound to the muropeptide after cleavage by Pronase E; **D33**, dimer muropeptide of a M3 cross-linked with a M3; **D34**, dimer muropeptide of a M3 l,d-cross-linked with a M4; **D34** ^**G**^, dimer muropeptide of a M3 cross-linked with a M3^G^ (Glc*N*Ac-Mur*N*ac-l-Ala-d-Glu-meso-DAP-Gly); **D43**, dimer muropeptide of a M4 d,d-cross-linked to a M3; **D44**, dimer muropeptide of a M4 cross-linked with a M4; **D44N**, dimer muropeptide of a M4 cross-linked with a M4N (anhydrous in the Mur*N*ac).

The enzymatic specificity of AP3 endolysin was examined *in vitro* using purified PG as a substrate with previously described methodology^31^. The chromatograms (Fig. 5A) demonstrated AP3gp15 muropeptide profiles similar to those obtained after lysozyme treatment suggesting similar specificity of both enzymes. Further confirmation by MS analysis of all major peaks present in both samples (Fig. 5B) identified identical products. Thus, comparable to lysozyme, AP3gp15 cleaves *β*-1,4-glycosidic bond of PG and releases Glc*N*Ac and Mur*N*Ac. These results confirm that AP3 endolysin is, in fact, a lysozyme, not a lytic transglycosylase.

### Activity and cytotoxicity analysis of recombinant AP3 endolysin

The muralytic activity of AP3 endolysin was assessed on permeabilized Gram-negative strains according to a standardized assay methodology^32^. The AP3gp15 turned out to be two times stronger than the commercially available lysozyme (Table 2). This was consistent for all tested strains, including *E*. *coli*, *K*. *pneumoniae*, *P*. *aeruginosa*, *B*. *cenocepacia* and *S*. *enterica* Typhimurium. No lytic activity was detected against Gram-positive *Staphylococcus aureus* and *S*. *epidermidis* strains. To verify that Glu101 is a key residue of AP3gp15 catalytic centre, the site-directed mutagenesis was performed and the Glu101 has been converted to an alanine (Glu101Ala). The muralytic activity of modified enzyme tested on *P*. *aeruginosa* PAO1 permeabilized cells, dropped down from 14,710 U/mg for wild-type version to 90 U/mg for protein mutant, reducing the lytic activity of 99.4%.

**Table 2:** The muralytic activity of phage AP3 endolysin and commercially available lysozyme (Sigma Aldrich, Poland) against selected bacterial strains.

Bacterial strain	AP3 endolysin (U/mg)	Commercial lysozyme (U/mg)
*Escherichia coli* (ATCC 25922)	14,120	7,400
*Klebsiella pneumoniae* (ATCC 700603)	11,970	6,200
*Pseudomonas aeruginosa PAO1* (ATCC 15692)	14,710	8,250
*Salmonella enterica serovar* Typhimurium LT2	12,690	6,800
*Burkholderia cenocepacia 7780*	9,000	3,600
*Staphylococcus aureus* ATCC 6538	0	0
*Staphylococcus epidermidis* ATCC 35983	0	0

The endolysin showed to be relatively stable at low temperatures but heat-sensitive. It retained > 95% activity after 1 week storage at −20 °C or 4 °C, at the concentration of 0.5 mg/ml in PBS buffer (pH 7.4). After 1 week at RT, the protein started to precipitate but still retained 70% residual activity. Ten minutes incubation at higher temperatures: 30 °C, 40 °C, 50 °C, 60 °C resulted in a decrease of around 2%, 25%, 80% and 92% of its hydrolytic activity, respectively. The incubation for 10 minutes at 80 °C caused a complete inactivation. The pH optimum was in the range of 7–9. The activity dropped at pH 6 and 10 by 10% and 25%, respectively. Low pH (pH = 5) reduced the activity to 58%. The cytotoxicity of AP3gp15 on mammalian cells was also evaluated and 50 µg/ml of the enzyme exhibited no adverse effect on A549 and THP-1 cells. After 48 h of incubation, no changes in cell viability and number were observed in comparison to endolysin free buffer control (data not shown).

## Discussion

Phage endolysins have a large, but yet unexplored potential as antimicrobials, due to poor structural and biochemical characterization. The annotation of endolysin encoding genes and their qualification to a specific enzymatic group are generally based on the amino acid sequence similarity to known proteins. Lack of a large reference library results, among others, in a false classification of newly characterized enzymes. One of the examples is the phage lambda endolysin which operates in the databases and in numerous publications as lysozyme, but in fact, has lytic transglycosylase activity^29,33^. The experimental verification of structural features and enzymatic specificity is a crucial step for prospective enzyme modification and further application. The thorough characterization of representative members is a key element in the case of conservative domains of unknown function (DUFs) which represent 20% of all currently annotated protein domains in Pfam database. The data obtained from the first member usually defines the primary characteristics of the entire protein family allowing a better understanding of the biology/function of many organisms.

In the present study, we describe a modular endolysin (rare for Gram-negative infecting phages) originating from *B*. *cenocepacia* AP3 phage. The domains arrangement of AP3gp15 (CBD at the N-terminus and EAD at the C-terminus) is characteristic for all eight endolysins originating from Gram-negatives specific phages with confirmed modular structure^10,12–15,34^. The AP3gp15 CBD element revealed a high structural similarity to CBDs of Zn-dependent amidase from *C*. *acetobutylicum* and *Pseudomonas* phage phiKZ endolysin. The latter domain has a high-affinity to Gram-negatives PG and is able to increase an enzymatic activity of phiKZ endolysin^23^. It should be noted that genomes of both phages: phiKZ (giant myovirus, 280,334 bp) and AP3 (*Peduovirinae;* 36,499 bp) belong to entirely different taxonomic lineages. Moreover, CBD of AP3 endolysin shows significant similarity to almost two and a half thousand homologues (phages and bacteria) in genome databases including a recently described endolysin from *Salmonella* phage 10^15^. Therefore, AP3gp15-like CBDs seem to be universal in general, irrespectively to the origin. It stays in contrast to endolysins encoded by Gram-positives specific phages, where CBDs demonstrate a large sequence diversity, and genus or even species specificity^4,35^.

The structural alignment highlights the similarity of DUF3380 of AP3gp15 to proteins having an activity of lysozyme and lytic transglycosylase (phage lambda endolysin). The catalytic mechanism of a vast majority of lysozymes relies on two residues: Glu and Asp. Catalytic centers of canonical lysozymes derived from T4 and P21 phages are based on the Asp residue separated in a sequence by eight residues relative to catalytic Glu. Although, the AP3gp15 also contains Asp at analogous sequence position (Asp110), at the structural level this residue is not equivalent. In T4 and P21 lysozymes, the residue forms the active site whereas in AP3gp15 it is positioned away from the catalytic center. The crystal structure analysis suggests that only a single catalytic residue (Glu101) is present at the active site of AP3 endolysin. Moreover, the comparative *in silico* analysis of AP3gp15 EAD homologues revealed that Asp is not a conserved residue in this protein family (present only in 123 out of 233 analyzed DUF3380 domains) what again might suggest an insignificant role in the catalysis. Instead of given Asp, other replacing amino acids: asparagine, threonine, serine, histidine, proline, tyrosine or alanine, can be found in sequenced DUF3380 homologues. Of functionally characterized member, a DUF3380 domain of *Salmonella* phage 10 endolysin has threonine residue at a position equivalent to 110 in AP3gp15 (Fig. 1A) and its active site most probably consists only of Glu101 residue, although no structure is available. Experimental data of AP3gp15 mutant Glu101Ala with a decline in activity by 99.4%, confirmed of Glu101 involvement in the catalytic reaction. However, based on the putative catalytic residues identified on other lysozymes by sequence and structure similarity, we were not able to identify a second catalytic residue. To date, only one phage endolysin (lysozyme Lys68) with Glu as a putative single catalytic residue, has been described, but no sequence homology to AP3gp15 is seen^10^. A single glutamic acid is characteristics for the catalytic centre of GEWL group of lysozymes^20^ and lytic transglycosylases^36^. Although the structural comparison of AP3gp15 EAD suggests a closer similarity to phage lambda endolysin (lytic transglycosylase) rather than GEWL, the biochemical analysis showed lysozyme-like cleavage products of PG digestion by AP3gp15. Similar results showing a lysozyme cleavage specificity were obtained for a related modular endolysin derived from *Salmonella* phage 10 by Rodríguez-Rubio and co-workers^15^. This indicates that DUF3380 has a HEWL-like fold, but a novel type of catalytic center basing on one residue as in GEWL and lytic transglycosylase. The structural similarity of the catalytic domain AP3gp15 to other glycosidases allows speculating on the catalytic mechanism. The substrate binding and related distortion of the cleaved bond, the formation of a covalent intermediate and the electrophilic movement of C-1 carbon along the reaction coordinate are the canonical basis^30^. HEWL was the first enzyme for which the three-dimensional structure was determined by X-ray diffraction^37^ and currently it is the best structurally characterized glycosidase in terms of the catalytic mechanism and substrate binding. The cleavage is preceded by the binding of PG hexasaccharide unit with concomitant distortion within Mur*N*Ac. Mur*N*Ac unit in the -1 (Glu) subsite adopts a half-chair conformation, thereby forming a kink in the oligosaccharide between sites -1 and + 1 (Glu and Asp). Glu35 of HEWL transfers a proton to the O1 position while the negative charge on Asp52 stabilizes the positively charged oxonium ion intermediate^30,38,39^. Despite the catalytic domain of AP3 endolysin being much larger than HEWL and the core of AP3gp15 aligns well with HEWL, the core of AP3gp15 contains additional protrusions which function is unknown. Although, the significantly different binding mode to HEWL is unlikely, the substrate binding surface of AP3gp15 may be extended in comparison to HEWL. If so, EAD of AP3 endolysin (DUF3380) could be the largest catalytic subunit described to date among lysozymes. The broad distribution and conservative character of DUF3380 domains in phages and bacteria advocate its important function. Moreover, the lack of amino acid sequence homology between AP3 endolysin and other glycosidases may reflect evolutionary convergence. That would imply that DUF3380-like and other classes of muralytic glycosidases had aroused in nature independently in different ancestral phages/bacteria as a result of an evolutionary trend in the development of peptidoglycan-degrading proteins. Particularly interesting is the fact, that despite differences in the known structures of lysozymes, the enzymatic specificity remains the same.

The AP3gp15 exhibits almost twice higher muralytic activity comparing to commercially available HEWL lysozyme, both tested on previously permeabilized bacterial cells. Compared to other endolysins derived from Gram-negatives specific phages, the AP3gp15 was classified as a medium effective enzyme, having 65-fold lower than the strongest 10gp110, to 6-fold higher activity comparing to the weakest KP32gp15^10,13–15,31,34^. Similarly to others, the AP3 endolysin shows a broad spectrum of activity against Gram-negative strains, but no PG-degrading properties with respect to Gram-positives^10,13,14,31,40,41^. This is consistent with the differences in PG structure. The thick PG of Gram-positive bacteria varies significantly in the peptide composition, crosslinks, and modifications of the glycan chain, whereas the PG of Gram-negatives is conserved having 1-3 layers of A1γ chemotype^42^. Like the vast majority of endolysins derived from Gram-negative specific phages, the AP3gp15 is not able to lyse intact cells and for this purpose requires the combination with the outer membrane permeabilizers or the binding to specific cationic peptide elements^9–11^. It is worth to mention that despite amino acid homology between AP3 and *Salmonella* phage endolysins, they differ in terms of bactericidal potential and thermostability. The AP3gp15 has 65-fold lower PG-hydrolysing efficacy than *Salmonella* phage endolysin (958,720 U/mg). Moreover, in contrast to the latter one, the AP3gp15 is unstable above 60 °C. The stability of *Salmonella* phage endolysin is probably related to the presence of disulphide bridges (DiANNA 1.1 web server prediction)^43^ which is absent in AP3gp15. The accurate determination of protein thermostability is an important issue in protein characterization and allows for the rational optimization of bioprocesses and for prospective protein modification to increase their thermal resistance^44–46^. Possibly, the introduction of disulphide bridges guided by *Salmonella* phage endolysin into AP3gp15 would increase its stability and thus the utility for industrial application. No adverse effect on eukaryotic cell lines (lack of cytotoxicity) promotes the AP3gp15 potential as an antimicrobial agent, as well.

Summarizing, *in silico* comparative analysis of AP3gp15 CBD and EAD (DUF3380) domains demonstrated unexpectedly a widespread occurrence within phages and bacteria and to a lesser extent also among archaea. The conserved character and wide distribution of both domains suggest their important function and evolutionary ancient origin. Therefore, the first report of DUF3380 domain structure and experimental characterization of its enzymatic activity presented in this study provides a significant contribution to the understanding of this widespread protein family with muralytic properties.

## Methods

### *In silico* analysis of AP3gp15

*In silico* analysis of AP3 endolysin included comparative protein sequence homology analysis, conserved domains recognition (NCBI’s BLASTP, HMMER, Phyre2, HHpred) and predicted physicochemical parameters (ExPASy ProtParam) using methodology described elsewhere^47–50^.

### Recombinant endolysin preparation

The AP3 endolysin (AP3gp15) sequence was amplified from AP3 genomic DNA by PCR using Pfu DNA polymerase (Thermo Fischer Scientific) and specific primers: forward: 5′-ATGTATAAAACCCTGCGCCTCGGCG-3′, 10 pM; reverse: 5′-CGCGGCCGCCCGGCTGTAGCGATCG-3′, 10 pM. The PCR product was cloned into commercially available pEXP5-CT/TOPO expression vector (Thermo Fischer Scientific) according to the manufacturer’s instruction. The construct was verified by sequencing and subsequently transformed into *Escherichia coli* BL21-AI (Thermo Fischer Scientific) expression strain. The culture was performed in LB medium at 37 °C with shaking (200 rpm) to achieve of mid-exponential-phase (OD600 ~ 0,6). Next, the expression was induced with L-arabinose (to a final concentration of 50.2%) and the incubation was continued at 20 °C for 18 hours. Cells were pelleted at 6000 x g, resuspended in lysis buffer (500 mM NaCl, 20 mM NaH_2_PO_4_, pH 7.7) and disrupted by the combination of freeze-thawing (−80 °C/RT; three times) and sonication (5 cycles of 15 s pulse and 30 s rest, Bandelin Sonopuls). Subsequently, the lysate was clarified at 15000 x g and recombinant protein was purified from filtered supernatant on NGC Medium Pressure Chromatography Systems (Bio-Rad) combined with Bio-Scale Mini Profinity IMAC Cartridges (Bio-Rad). The fractions containing recombinant protein were eluted (500 mM NaCl, 20 mM NaH_2_PO_4_, 500 mM imidazole, pH 7.7) and pooled. Protein was dialyzed against phosphate-buffered saline (PBS; pH 7.4) using Spectra/Por ® Float-A-Lyzer (Spectrum Laboratories, Inc. Rancho Dominguez, CA, USA). The concentration of purified recombinant enzyme was then determined fluorimetrically using Qubit® Protein Assay Kit, (Thermo Fisher Scientific). Seleno-methionine (SeMet) labelled endolysin was expressed in PASM-5052 medium containing 10 µg/ml methionine, 125 µg/ml SeMet, and 100 nM vitamin B12 according to Studier’s method^51^.

### Determination of muralytic potential

The PG degrading activity and antibacterial spectrum of AP3gp15 were determined on five Gram-negative strains: *E*. *coli* (ATCC 25922), *K*. *pneumoniae spp*. *pneumoniae* (ATCC 700603), *P*. *aeruginosa* PAO1 (ATCC 15692), *S*. *enterica* serovar Typhimurium LT2 (food isolate, Centre of Food and Microbial Technology of the KU Leuven, Belgium), *B*. *cenocepacia* lineage IIIA (clinical isolate 7780, PCM 2854) and two Gram-positive strains: *S*. *aureus* (ATCC 6538), and *S*. *epidermidis* (ATCC 35983) (Table 2). In Gram-negative strains the outer membrane was removed by 45 min incubation in chloroform-saturated 50 mM Tris–HCl buffer (pH 7.7), according to Lavigne *et al*.^52^ methodology. The muralytic activity of AP3 phage endolysin was assayed as described by Briers *et al*.^32^. 270 µl of bacterial culture was combined with 30 µl of endolysin (final concentration range of 50 ng/ml – 200 ng/ml). As a control, the same concentrations of commercially available lysozyme from chicken egg white (Sigma Aldrich) was used. The kinetic drop in culture turbidity in the presence of endolysin or commercial lysozyme was measured spectrophotometrically using microplate reader (Asys UVM340). The muralytic activity was quantified using a standardized method developed by Briers *et al*.^32^, where 1 unit corresponds to the amount of endolysin resulting in an OD_600_ decrease in culture turbidity of 0.001/min.

### Determination of AP3 phage endolysin stability

Storage stability in PBS buffer (pH 7.4) was determined by incubating the endolysin for 1 week at RT and 1 month at 4 °C and −20 °C. The thermostability of enzyme was determined in PBS (pH 7.4) by 10 min incubation at 30 °C, 40 °C, 50 °C, 60 °C and 70 °C. The pH stability was defined in the universal buffer (50 mM KCl, 10 mM KH_2_PO_4_, 10 mM Na-citrate and 10 mM H_3_BO_4_) adjusted to different pH (from 3 to 10 using) by concentrated NaOH or HCl. In each case, the residual activity was determined by monitoring the PG degrading activity against permeabilized *P*. *aeruginosa* PAO.

### PG isolation and analysis of endolysin cleavage specificity

*E*. *coli* cells from 0.2 l overnight culture were pelleted at 4,500 x g and resuspended in 5 ml of PBS. An equal volume of 10% SDS was added and the sample was incubated in a boiling water bath with vigorous stirring for 4 h. Following the sample was further stirred overnight at RT. The insoluble fraction (PG) was pelleted at 400,000 x g, 15 min, 30 °C (TLA-100.3 rotor; OptimaTM Max ultracentrifuge, Beckman). SDS was washed out and the PG was treated with Pronase E 0.1 mg/ml at 60 °C for 1 h and further boiled in 1% SDS for 2 h to stop the reaction. The sacculus was resuspended in 200 μl of 50 mM sodium phosphate buffer pH 4.9 and digested overnight with 30 μg/ml muramidase (Cellosyl) or with 0.4 mg/ml of AP3gp15 in Tris-HCL 20 mM buffer pH 8.0, 1 mM MgCl_2_ and 1 mM ZnCl_2_. Samples were incubated at 37 °C. PG digestion was stopped by 5 min incubation in a boiling water bath. Coagulated protein was removed by centrifugation. The supernatants were mixed with 150 μl 0.5 M sodium borate pH 9.5, and subjected to reduction of muramic acid residues into muramitol by sodium borohydride treatment (10 mg/ml final concentration, 30 min at RT). Samples were adjusted to pH 3.5 with phosphoric acid. Chromatographic analyses of muropeptides were performed on ACQUITY Ultra Performance Liquid Chromatography (UPLC) BEH C18 column (130 Å, 1.7 μm, 2.1 mm by 150 mm; Waters), and peptides were detected at Abs. 204 nm using ACQUITY UPLC UV-Visible Detector. Muropeptides were separated using a linear gradient from buffer A (PBS 50 mM, pH 4.35) to buffer B (PBS 50 mM, pH 4.95 methanol 15% (v/v)) in 20 min, and flow 0.25 ml/min. The peaks from chromatogram that did not show similarity to any of previously described muropeptides were considered as background noise and removed from the chromatogram. Eluting peak samples were analyzed with Agilent 6550 iFunnel Q-TOF LC/MS System (Agilent Technologies) using an organic separation method^53^.

### Crystallization, X-ray data collection, and structure determination

Native and SeMet-labelled AP3gp15 was concentrated to 15 mg/ml in a buffer containing 0.01 M Tris-HCl (pH 8.0), 0.05 M NaCl and 5 mM DTT. Crystallization screening was performed by sitting-drop vapour diffusion method using1:1 ratio of protein and reservoir solution at RT. A thin needle-like crystal of SeMet-AP3gp15 was obtained from a solution containing 0.1 M Tris-HCl (pH 8.0), 0.15 M NaCl and 8% w/v PEG 6000 (ProPlex, Molecular Dimension). Native AP3gp15 crystals were obtained from 0.1 M SPG (pH 7.0), 25% w/v PEG 1500 (PactPremier, Molecular Dimension). Crystals were cryoprotected in 25% glycerol in the mother liquor and flash cooled in liquid nitrogen.

The diffraction data were collected at beamline P11 PETRA III at DESY. A native data set extending to 1.72 Å resolution was collected using 0.9184 Å wavelength, while a data set for SeMet-labelled protein crystals were collected at the peak wavelength (0.9795 Å) and extended to 2.42 Å resolution. Data were integrated and scaled using XDS^54^ and SCALA^55^ from the CCP4 suite, respectively. The native crystals belonged to space group H32, and the Matthews coefficient suggested the presence of two monomers in the asymmetric unit. SeMet-labelled crystals belonged to space group P1211 with four monomers in the asymmetric unit.

The initial structure was determined by single-wavelength anomalous dispersion (SAD) using AutoSol program included in the PHENIX suite^56^. The model was constructed and partially refined in an automated mode using AutoBuild and Buccaneer^57^ from PHENIX and CCP4 suites, respectively. Initial, partially refined structure was used as a search model for molecular replacement using native data and performed with Phaser^58^. Refinement was performed with REFMAC5^59^ to complement manual rebuilding using COOT^60^. R_free_ was used to monitor the refinement strategy. Water molecules were added in 2Fobs – Fcalc map at densities above 2σ at reasonable sites upon visual inspection. Data processing and refinement statistics are summarized in Table 1. The geometry of the final model was analyzed with Molprobity^61^. Structural similarity searches were carried out using Dali server^25^ and Z scores of > 10 were considered as significant.

### Site-directed mutagenesis

To establish the putative catalytic residue within the endolysin sequence, the active site mutation (Glu101Ala) was introduced. Two overlapping specific primers: forward 5′-GCCGTCAATGAGGTTGCATCGAAAGGTGCCGGG-3′ (10 pM) and reverse 5′-CCCGGCACCTTTCGATGCAACCTCATTGACGGC-3′ (10pM) with mutation basepairs underlined, were applied. The previously constructed expression vector (pEXP5-CT/TOPO plasmid containing the AP3gp15 DNA) was mutated using above primers and the GeneArt® Site-Directed Mutagenesis System from Thermo Fischer Scientific. The mutant was prepared, purified using the same protocol as for the wild type of AP3gp15. The PG muralytic activity of the purified AP3gp15 Glu101Ala mutant was quantified using permeabilized *P*. *aeruginosa* PAO1 cells, and protocol described below in “*determination of muralytic potential*” section.

### Determination of endolysin cytotoxicity

Determination of endolysin cytotoxicity against human cells has been chosen as preliminary safety evaluation. Cytotoxicity of AP3gp15 was examined with respect to lung carcinoma epithelial cell line A549 (ATCC CCL-185) and acute monocytic leukaemia cell line THP-1 (ATCC TIB-202) using trypan blue assay. Endolysin suspended in PBS buffer pH 7.4 was added to exponentially growing cells (1 × 10^5^ cells/ml) to a final concentration of 50 µg/ml. Cells were incubated for 24 h and 48 h in a suitable medium: DMEM (for A549) or RPMI 1640 (for THP-1), both supplemented with 2 mM glutamax, 1% antibiotic–antimycotic, and 10% heat-inactivated fetal bovine serum (all media and supplements from Gibco BRL, Thermo Fischer Scientific). Cell viability was determined by trypan blue staining (0.4% in PBS; Sigma Aldrich, Germany) and compared to a number of cells in the control (PBS treatment only).

### Data availability

The protein sequence of AP3gp15 can be found in GenBank under accession number: AKA61137.1. Coordinates and structure factors for the AP3gp15 crystal structure have been deposited in the Protein Data Bank under accession code PDB 5NM7. Other data supporting this study are available from the corresponding authors upon reasonable request.
